# Porous Osteoplastic Composite Materials Based on Alginate–Pectin Complexes and Cation-Substituted Hydroxyapatites

**DOI:** 10.3390/polym17131744

**Published:** 2025-06-23

**Authors:** Galina A. Davydova, Inna V. Fadeeva, Elena S. Trofimchuk, Irina I. Selezneva, Muhriddin T. Mahamadiev, Lenar I. Akhmetov, Daniel S. Yakovsky, Vadim P. Proskurin, Marco Fosca, Viktoriya G. Yankova, Julietta V. Rau, Vicentiu Saceleanu

**Affiliations:** 1 Federal State Institution of Science Institute of Theoretical and Experimental Biophysics of the Russian Academy of Sciences (ITEB RAS), Institutskaya St., 3, Pushchino, Moscow 142290, Russia; davidova_g@mail.ru (G.A.D.); selezneva_i@mail.ru (I.I.S.); yackowsckay@mail.ru (D.S.Y.); vadimapro@bk.ru (V.P.P.); 2 Federal State Budgetary Institution of Science A.A. Baikov Institute of Metallurgy and Materials Science of the Russian Academy of Sciences (IMET RAS), Leninsky Ave, Moscow 119334, Russia; ifadeeva@imet.ac.ru; 3 Faculty of Chemistry, Federal State Budgetary Educational Institution of Higher Education “Lomonosov Moscow State University”, Lenin Hills, 1, Bld. 3, GSP-1, Moscow 119991, Russia; trofimchuk@vms.chem.msu.ru; 4 Federal State Budgetary Institution of Science P.N. Lebedev Physical Institute of the Russian Academy of Sciences, 53, Leninsky Prospekt, Moscow 119991, Russia; mahamadievm@lebedev.ru; 5 Scriabin Institute of Microorganisms, Institutskaya St., 7, Pushchino, Moscow 142290, Russia; lenarakhmetov@pbcras.ru; 6 Istituto di Struttura della Materia, Consiglio Nazionale delle Ricerche (ISM-CNR), Via del Fosso del Cavaliere, 100, 00133 Rome, Italy; marco.fosca@ism.cnr.it; 7 Department of Analytical, Physical and Colloid Chemistry, Institute of Pharmacy, I.M. Sechenov First Moscow State Medical University, Trubetskaya 8, Build. 2, Moscow 119048, Russia; yankova_v_g@staff.sechenov.ru; 8 Faculty of Medicine, University Lucian Blaga Sibiu, 2A Lucian Blaga Street, 550169 Sibiu, Romania; vicentiu.saceleanu@ulbsibiu.ro

**Keywords:** porous composite material, sponge, alginate, pectin, manganese-substituted hydroxyapatite, zinc-substituted hydroxyapatite

## Abstract

Novel three-dimensional porous composites of alginate–pectin (A/P) with zinc- or manganese-substituted hydroxyapatites (A/P-ZnHA and A/P-MnHA) were synthesized via lyophilization and calcium cross-linking. Powder X-ray diffraction and infrared spectroscopy analyses confirmed single-phase apatite formation (crystallite sizes < 1 µm), with ZnHA exhibiting lattice contraction (*c*-axis: 6.881 Å vs. 6.893 Å for HA). Mechanical testing revealed tunable properties: pristine A/P sponges exhibited an elastic modulus of 4.7 MPa and a tensile strength of 0.10 MPa, reduced by 30–70% by HA incorporation due to increased porosity (pore sizes: 112 ± 18 µm in the case of MnHA vs. 148 ± 23 µm-ZnHA). Swelling capacity increased 2.3–2.8-fold (125–155% vs. 55% for A/P), governed by polysaccharide interactions. Scanning electron microscopy investigation showed microstructural evolution from layered A/P (<100 µm) to tridimensional architectures with embedded mineral particles. The A/P-MnHA composites demonstrated minimal cytotoxicity for the NCTC cells and good viability of dental pulp stem cells, while A/P-ZnHA caused ≈20% metabolic suppression, attributed to hydrolysis-induced acidification. Antibacterial assays highlighted A/P-MnHA′s broad-spectrum efficacy against Gram-positive (*Bacillus atrophaeus*) and Gram-negative (*Pseudomonas protegens*) strains, whereas A/P-ZnHA targeted only the Gram-positive strain. The developed composite sponges combine cytocompatibility and antimicrobial activity, potentially advancing osteoplastic materials for bone regeneration and infection control in orthopedic/dental applications.

## 1. Introduction

The development of regenerative medicine requires the creation of new biologically active osteoplastic matrices capable of filling bone defects and stimulating de novo bone tissue regeneration. Within the framework of this concept, matrices should fill the tissue defect, guide cell growth, stimulate bone formation, and gradually resorb, ultimately being completely replaced by newly formed tissue [1,2]. The main requirements for such matrices are biocompatibility, ability to support adhesion, growth and osteogenic differentiation of cells, and to ensure that the timing of resorption is coordinated with the rate of defect replacement by the newly formed tissue. These characteristics of matrices are in direct dependence on their chemical composition and pore space microstructure [3,4,5]. Porous mineral–polymer materials are most suitable for surgical use, as their excellent deformability allows them to fully fill defect spaces.

Hydroxyapatite Ca_10_(PO_4_)_6_(OH)_2_ (HA), which is the main mineral component of bone tissue, is often used as a mineral component to stimulate bone formation. However, HA-based large-crystalline ceramics resorb slowly so that inclusions of artificial material can be found in bone after many years [6]. Isomorphic substitutions of calcium for other cations (in particular, manganese (Mn) or zinc (Zn)) create localized stresses in the HA lattice, contributing to an increase in the rate of material resorption. Among many known substituent cations, magnesium, manganese, zinc, strontium, and iron ions have long attracted the attention of researchers for their inclusion in HA-based bioceramics due to their therapeutic effects [7].

Sodium alginate and pectin are natural polysaccharides that can form high-water-content hydrogels that mimic the natural intercellular matrix [8]. Alginate is a polyanionic hydrophilic natural polymer derived from marine brown algae. It is a heteropolymer, formed by two polyuronic acid residues (D-mannuronic acid and L-guluronic acid) in different proportions depending on the particular algal species. The addition of polyvalent metal salts to sodium alginate solution leads to the formation of insoluble gels of these metal alginates, which are often used to partially cross-link materials containing alginate [9]. Alginate has low toxicity, is biocompatible [10], and is widely used in medical applications such as tissue engineering, dentistry, and wound dressing [11]. Pectin is a polyanionic hydrophilic polymer extracted from fruit and vegetable cakes and is a homopolymer composed mainly of galacturonic acid linked by α-1,4-glycosidic bonds [12]. It forms gels and is non-toxic, making it a key component in pharmaceutical products [13]. Due to its gel-forming properties, biocompatibility, and biodegradability, pectin finds applications in the creation of microcapsules for cell delivery [14], scaffolds for tissue regeneration, and dressings [15,16].

Previously, the authors of this paper developed and investigated hydrogels based on a mixture of polymers–alginate and pectin [17]. The aim of this study was to develop porous mineral–polymer composites based on alginate–pectin (A/P) complexes and cation-substituted hydroxyapatites (ZnHA and MnHA)-A/P-ZnHA and A/P-MnHA and to investigate their effects on the physicochemical (composition, microstructure, mechanical properties, swelling dynamics) and biological (biocompatibility, antibacterial activity) properties of porous mineral–polymer matrices.

To test the cytotoxicity of A/P-MeHA composites, the fibroblasts of the NCTC clone L929 cell line were used. The viability of dental pulp stem cells (DPSCs) was accessed. A Gram-positive bacteria strain of *Bacillus atrophaeus* B-723 (*B. atrophaeus*) and of Gram-negative *Pseudomonas protegens* (*P. protegens)* were used to investigate the bactericidal properties of the prepared materials.

## 2. Materials and Methods

### 2.1. Synthesis of Cation-Substituted HAs

ZnHA and MnHA were synthesized as previously described in [18]. The synthesis was carried out according to reaction (1):

10xCa^2+^ + 10(1 − x)M^2+^ + 6PO_4_^3−^ + yOH^−^ + zCO_2_ → Ca_9.5_M_0.5_(PO_4_)_6_(OH)_y_(CO_3_)_z_ (1)

where M = Zn; Mn; y = 2 − 0.5z.

Briefly, 380 mL of a 1 M calcium nitrate solution was mixed with 20 mL of a 1 M zinc or manganese acetate solution in a reactor equipped with a propeller stirrer. Subsequently, 100 mL of a 25% aqueous ammonia solution was added. While stirring at 400 rpm, 400 mL of a 0.6 M diammonium phosphate solution was introduced dropwise. The mixture was stirred for 1 h and then aged for 24 h at room temperature. The resulting precipitate was collected by vacuum filtration using a Büchner funnel, dried in a desiccator at 110 °C for 12 h, and manually disaggregated in an agate mortar. Finally, the powder was calcined at 400 °C to eliminate residual by-products, including ammonium nitrate and adsorbed water.

### 2.2. XRD Analysis

The phase composition of the obtained phosphates was determined with a Shimadzu 6000 X-ray diffractometer (Kyoto, Japan) in reflection mode using Cu Kα radiation after calcination for 1 h at 900 °C.

### 2.3. FT-IR Spectroscopy

IR transmission spectra were recorded on a Nicolet Avatar-330 FT-IR spectrometer (Thermo Fisher Scientific, Waltham, MA, USA) in the range 4000–400 cm^−1^ with a resolution of 0.9 cm^−1^. The samples were investigated in mixtures with potassium bromide. The spectra were analyzed based on the literature data [19,20,21].

### 2.4. Alginate/Pectine-Metal Substituted HA Composite (A/P-MeHA) Preparation

To obtain bulk porous composite materials, 0.5% of HA or MeHA was introduced into 2% A/P solution with a ratio of 1:1. The resulting mixture was then whipped, using a top-driven stirrer, at a stirrer speed of 1500 min^−1^. The sponge mass was frozen at −40 °C and further dried in an Iney 4 lyophilic dryer (SKB BP, Pushchino, Moscow, Russia). The dried samples were placed in 3% CaCl_2_ solution, and the bulk materials were removed from the solution after 5 min and dried in air at T = 22 °C.

### 2.5. Raman Spectroscopy

Raman spectra were collected using a Raman microscope (Renishaw, Gloucester, UK) [21] equipped with a laser diode with a wavelength of 785 nm and a power of 45 mW at the cuvette inlet. Raman spectra were recorded in a backscattering mode. The laser beam was focused on the surface of the sponges using an NPlan 50/0.50 lens with a focal length of 8 mm (Leica, Wetzlar, Germany). For each sample, an exposure time of 20 s and a 10 multiple scan were set in order to collect the spectrum, resulting in a 200 s scan for each recording. The recorded spectra were processed by means of the Renishaw software (RenishawFixture-Builderx64, Renishaw, Gloucester, UK) and Origin 8.5.

### 2.6. SEM

The microstructure of the samples was studied using a scanning electron microscope (SEM) (Tescan Vega II, Tescan, Brno, Czech Republic) with an energy-dispersive X-ray spectrometer (EDS). The accelerating voltage of the electron gun was 17–21 kV. Elemental microanalysis was carried out by an energy-dispersive microanalysis system INCA Energy 300 (Oxford Instruments, Abingdon, Oxfordshire, UK).

### 2.7. Swelling Measurements

The swelling degree of A/P sponges containing ZnHA or MnHA was evaluated by monitoring their weight change upon immersion in physiological solution (0.9% NaCl) over time. The sponges were incubated at different set intervals (1, 3, 6, 12, 24, 48, and 72 h), with their initial weight (*M*_0_) and wet weights (*M_X_*) recorded at each time point [22]. The swelling coefficient (*K*_s_, %) was calculated using the following equation:

(2) $$K_{s}=\frac{{\;M}_{x}-M_{0}}{M_{0}}*100$$

### 2.8. Mechanical Tests

Mechanical testing was performed on hydrated specimens (containing 10.5–12.5 wt.% water, as determined by thermogravimetric analysis) using a Z3-X500 Thümler universal testing machine (Thümler, Germany) equipped with a 50 N load cell (Nordic Transducer, Hauch & Bach ApS, Lynge, Denmark). All tests were conducted in air at 25 °C with a crosshead speed of 0.5 mm/min.

For tensile testing, rectangular strips with a working length of 30 mm and a width of 6.5 mm were prepared and subjected to uniaxial loading until rupture. The thickness of the samples was measured using a MCC-25 digital micrometer, accuracy ±0.001 mm (Etalon, China). Tensile strength was tested according to the ASTM D3574 Test E. Additionally, cyclic tension–compression tests (hysteresis evaluation) were performed on the same strip specimens.

For compression and cyclic compression–recovery tests (elasticity assessment), cylindrical specimens (15 mm diameter) were prepared. Compression properties were tested according to the ASTM D1056-20. Cyclic loading was applied with a stepwise deformation increment of 1% per cycle.

Each mechanical test was performed on 3 samples. The statistical data processing was carried out using a standard procedure, which included the calculation of the standard deviation values.

### 2.9. In Vitro Cell Tests

Postnatal human dental pulp stem cells were isolated from the rudiment of a third molar extracted for orthodontic indications, as described in [23]. Cells were grown in DMEM/F12 medium (PanEco, Moscow, Russia) supplemented with 10% fetal calf serum FBS (HyClone, Marlborough, MA, USA) in a humidified incubator at 37 °C and 5% CO_2_. The medium was changed after 24 h in primary cell culture. When 80% monolayer confluency was reached, cells were passaged in a 1:4 ratio using trypsin (0.25%)-EDTA solution (PanEco, Moscow, Russia). DPSCs of the fourth passage were used for the study.

Cell viability in the presence of sponges was analyzed by a direct contact method [17]. For this purpose, cells were seeded in the wells of a 24-well plate at a concentration of 25 thousand cells/cm^2^ in DMEM/F12 medium (1:1) supplemented with 10% fetal calf serum (FBS) and 100 U/mL penicillin/streptomycin and 2 mML-glutamine and cultured at 37 °C in 5% CO_2_ atmosphere. After 24 h, sterilized sponges made from pectin and sodium alginate blends with hydroxyapatite or MeHA were placed in the cells of a 24-well plate and cultured at 37 °C in a 5% CO_2_ atmosphere for 24 h.

DPSC viability was assessed after 1 day by differential fluorescent staining. Microphotography was performed on an Axiovert 200 inverted microscope (Zeiss, Oberkochen, Germany). The fluorescent dye SYTO 9 in the study mode λ_excit_ = 450–490 nm, λ_emiss_ = 515–565 nm stains DNA and RNA of living and dead cells green. The intercalating reagent propidium iodide in the study mode λ_excit_ = 546 nm, λ_emiss_ = 575–640 nm stains the nuclei of dead cells red.

The cytotoxicity of A/P-MeHAs was investigated using extracts prepared according to the requirements [24,25] in DMEM/F12 culture medium on fibroblasts of the NCTC clone L929 line using the MTT test.

### 2.10. Determination of Antibacterial Activity by Direct Contact Method

Antibacterial activity was tested as described in [26]. A strain of Gram-positive bacteria *B. atrophaeus* B-723 and a rhizosphere strain of Gram-negative bacteria *P. protegens* P4-2 were used to study the bactericidal properties of the prepared materials. The liquid LB medium was infected with biomass of the strain by microbial loop [26] in a test tube and incubated overnight on a rocker at 28 °C, after which a 50 µL drop was taken, rubbed on the surface of the agar medium, and the lawn was grown for 12 h. In the morning, a 10 mm by 10 mm square piece of sponge was introduced with tweezers onto the surface of agar LB medium on a Petri dish. The dishes were cultured at 28 °C for two days. After that, the areas of translucency on the lawn were evaluated as zones of suppression.

### 2.11. Statistical Analysis

Viability assay and microbial inhibition tests results were expressed as an average value of five measurements ± standard deviation (SD). Statistical significance tests between the groups were carried out by the one-way ANOVA test (OriginPro 8.5, OriginLab, Northampton, MA, USA). The resulting *p*-values were reported in figures as follows * = *p* < 0.05, ** = *p* < 0.01, *** = *p* < 0.001.

## 3. Results and Discussion

**Figure 1:**
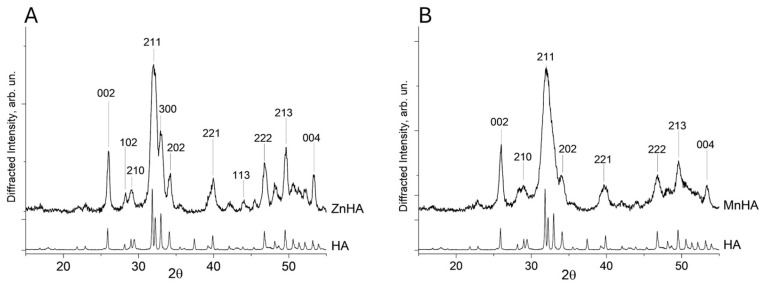
Diffractograms of ZnHA vs. HA (**A**) and MnHA vs. HA (**B**). The most intense Bragg′s reflections were labeled with their respective Miller indexes.

Analysis of the XRD results was conducted on both ZnHA and MnHA after calcination at 900 °C for 1 h. According to the literature, this thermal treatment promotes the formation of a pure and less amorphous HA phase [27,28]. The XRD outcomes confirmed that the synthesized ZnHA sample is characterized by a single-phase apatite structure (Figure 1A).

The diffraction peaks of the pattern collected on the ZnHA sample (Figure 1A) correspond to a pure hydroxyapatite phase (PDF card no. 09-0433). The lattice parameter *a* for both HA and ZnHA remained nearly identical (9.430 Å), whereas the parameter *c* decreased slightly from 6.893 Å (unsubstituted HA) to 6.881 Å (ZnHA). This contraction in the *c*-axis is consistent with previous reports [29] and can be attributed to the substitution of Ca^2+^ ions by Zn^2+^ in the crystal lattice.

In contrast, the MnHA diffractogram (Figure 1B) exhibited peak broadening, indicative of low crystallinity and a small particle size (<1 µm).

**Figure 2:**
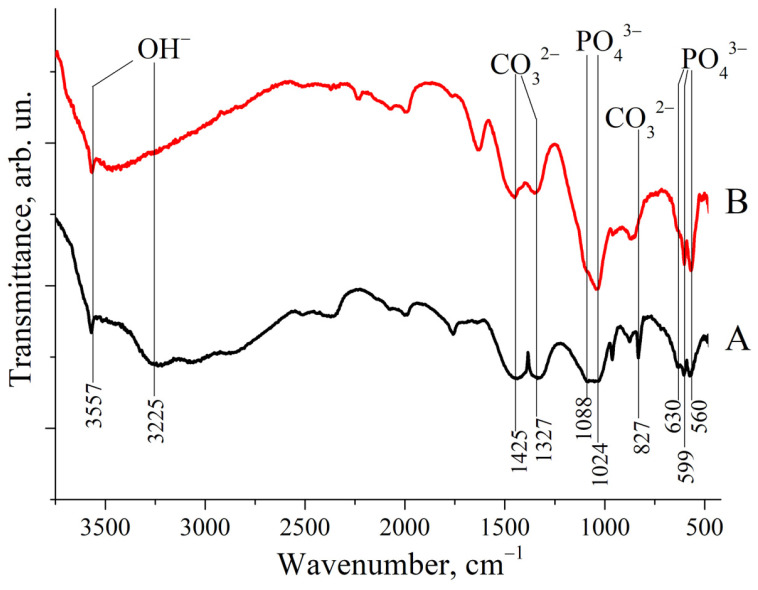
IR spectra of substituted HAs: (A) ZnHA; (B) MnHA. Band positions and attributions are labeled.

As shown in Figure 2, the IR spectra of both ZnHA and MnHA exhibit characteristic HA bands, including bands positioned around 1000 cm ^−1^ and a distinctive triplet in the 550–650 cm^−1^ region. Specific vibrational bands were observed at 560, 599, and 630 cm^−1^, corresponding to phosphate (ν_4_ $PO_{4}^{3-}$) and hydroxyl group vibrations at 3225 and 3557 cm^−1^. These IR spectroscopic findings support the previously reported XRD results. Additional bands at 827, 1327, and 1425 cm^−1^ were identified as carbonate ($CO_{3}^{2-}$) vibrational modes [30].

The presence of carbonate groups in the HA structure, consistent with previous reports [18], is attributed to synthesis under alkaline conditions in an ambient atmosphere containing CO_2_.

Several literature sources have reported comparisons between the IR spectra of pure (calcined) hydroxyapatite (HA) and those of HA synthesized via wet methods [27] or derived from natural sources [28]. The latter typically contains a higher proportion of water (H_2_O) and carbonate species (CO_2_, $CO_{3}^{2-}$), as evidenced by the presence of relatively strong absorption bands associated with OH^−^ and $CO_{3}^{2-}$ groups.

The FTIR spectra shown in Figure 2 exhibit relatively weak signals corresponding to OH^−^ stretching vibrations at 3225 and 3557 cm^−1^ and to $CO_{3}^{2-}$ modes at 827, 1327, and 1425 cm^−1^. A comparison between these IR spectra and those reported in the literature for HA suggests that both ZnHA and MnHA retain residual carbonate species (CHA) and embedded water. This is particularly evident in the MnHA spectrum.

**Figure 3:**
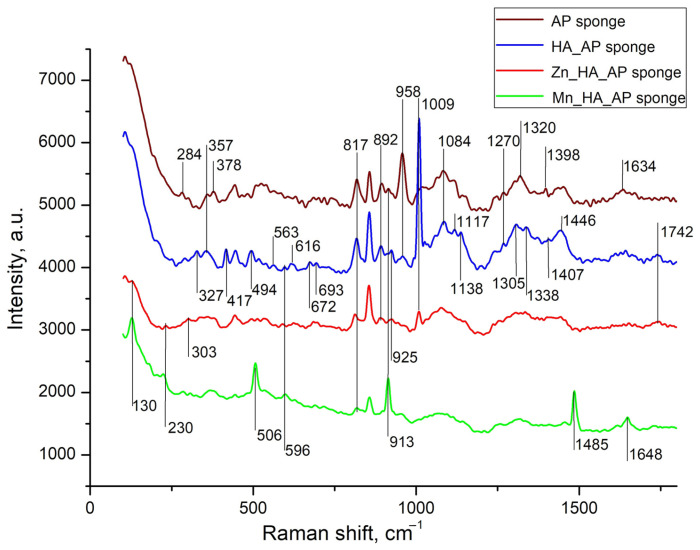
Raman spectra of A/P sponge (brown line), composites of A/P-HA sponge (blue line), A/P-ZnHA (red line) and A/P-MnHA (green line). The spectra are shifted along the vertical axis for better visualization. Raman shifts of most intense bands are labeled.

Raman spectroscopy is more suitable than IR spectroscopy for studying sponges containing metal phosphates, as it provides better-defined spectra for polymers [31]. The background part described by two Gaussians and apparently related to luminescence [17] was subtracted from the obtained spectra, after which the smoothing was performed, and the results are shown in Figure 3.

The Raman spectra comparison between pure alginate–pectin sponges and A/P-HA composites revealed vibrational bands characteristic of both components. Phosphate group vibrations appeared at 1010–960 cm^−1^ (ν $PO_{4}^{3-}$/$HPO_{4}^{2-}$ stretching modes) and 440–620 cm^−1^ (δ$PO_{4}^{3-}$/$HPO_{4}^{2-}$ bending modes). Several A/P bands exhibited significant shifts after HA incorporation: the 303 cm^−1^ band shifted to 327 cm^−1^ with increased intensity, while bands at 913, 1305, 1398, and 1634 cm^−1^ shifted to 925, 1338, 1407, and 1644 cm^−1^, respectively. The 958 cm^−1^ band showed substantial weakening, whereas the 1084 cm^−1^ band gained intensity. These modifications indicate strong interactions between the Ca^2+^-cross-linked AP matrix and HA nanoparticles.

It is worth underscoring that, while previous studies reported minimal compositional changes when incorporating calcium phosphate into sodium alginate [32], our A/P-HA system displayed distinct shifts. The -C-H deformation vibration at 1340 cm^−1^ shifted to 1307 cm^−1^, and the symmetric COO^−^ stretching vibration (characteristic of sodium alginate at ~1413 cm^−1^ [33]) shifted to 1446 cm^−1^. These changes reflect the replacement of Na^+^ by Ca^2+^ ions, which modifies the local charge density and coordination environment around the carboxylate groups [34,35].

The substitution of Ca^2+^ with Zn^2+^ or Mn^2+^ in HA induced further spectral modifications [36,37,38,39]. Both substitutions caused the disappearance of several AP bands (327, 357, 958, 1117, and 1270 cm^−1^) that were preserved in the A/P-HA composite. For MnHA, new features emerged, including the re-appearance of AP bands at 378 and 913 cm^−1^ and the appearance of a distinct Mn-O vibration at 506 cm^−1^ [39]. The ZnHA spectrum showed partial retention of the 1009 cm^−1^ (HA) and 1242 cm^−1^ (A/P) bands, suggesting incomplete Ca^2+^ substitution by Zn^2+^.

Metal-specific effects were particularly evident in the mannuronic acid region. While the A/P-HA composite showed a characteristic peak at 817 cm^−1^, this shifted to 811 cm^−1^ in ZnHA-containing sponges, indicating zinc′s ability to cross-link mannuronic acid residues [37,38]. In contrast, MnHA composites maintained this peak at 820 cm^−1^. Additional spectral changes included the appearance of weak bands at 989 and 1236 cm^−1^ in ZnHA sponges and strong new bands at 130, 506, 913, and 1485 cm^−1^ in MnHA sponges.

These observations can be explained by the different coordination geometries adopted by the metal ions. While Ca^2+^ forms cross-links through sp^2^ hybrid orbitals, the transition metals Zn^2+^ and Mn^2+^ utilize sp^3^ hybridization [40]. This difference in coordination chemistry accounts for the observed weakening of C-C, C-OH, and C-O bonds in the metal-substituted composites, as well as the distinct vibrational patterns observed in their Raman spectra.

**Figure 4:**
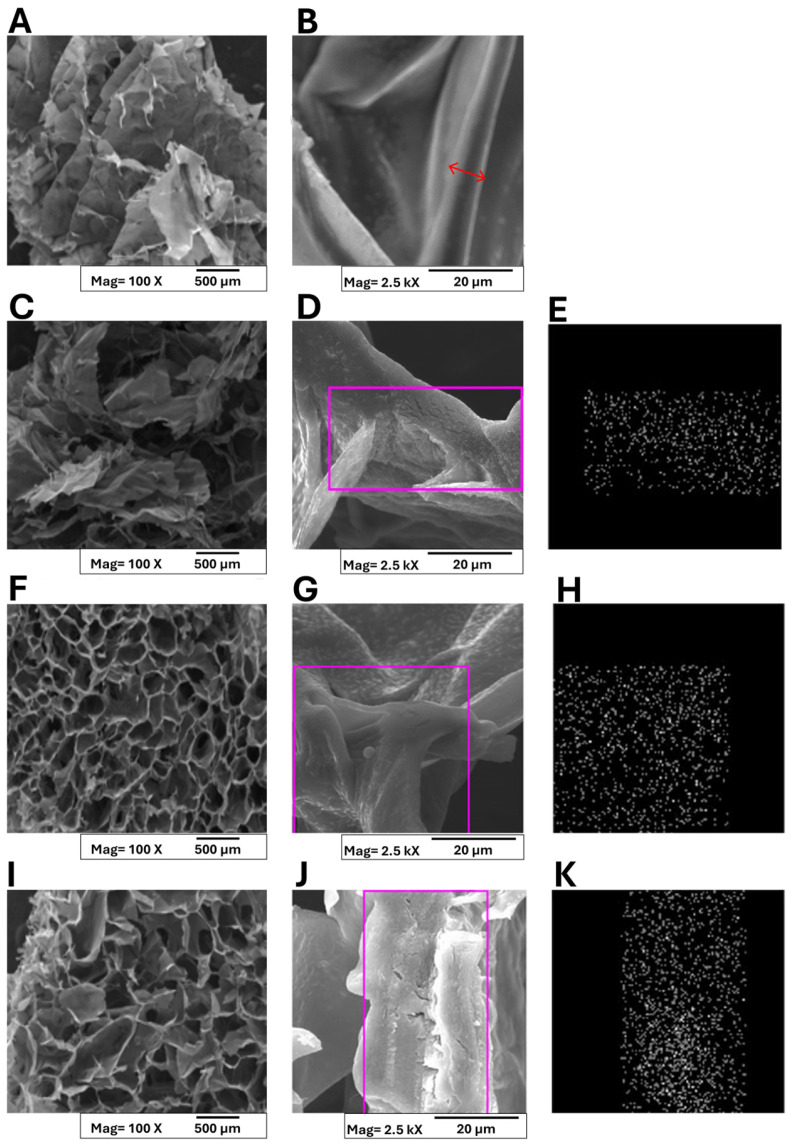
SEM micrographs and of Ca^2+^ ions distribution in polymer matrices collected on A/P (**A**,**B**); A/P-HA HA (**C**–**E**); A/P-MnHA (**F**–**H**); A/P-ZnHA (**I**–**K**). Panels (**E**,**H**,**K**) present EDS analyses highlighting the distribution of Ca^2+^ ions within the composite matrices, specifically in the selected regions of interest. A red arrow was superimposed to layer section (**B**) to highlight the layer thickness.

Conventional methods employed for CaCl_2_ addition to alginate solutions often result in asymmetric hydrogels due to rapid gelation kinetics [41]. To overcome this limitation, in this research, an alternative strategy involving post-lyophilization cross-linking of sponges with calcium salts, which yielded homogeneous structures, was employed. Figure 4 presents the macro- and micro-morphological features of both pristine alginate and pectin (A,B) and A/P-MeHA composite sponges (C-K).

At a macro-scale, all samples exhibited three-dimensional architectures with interconnected pore networks. The pure alginate–pectin matrix displayed a characteristic layered organization with individual layer thicknesses below 100 μm (Figure 4B). A red arrow was superimposed on the layer section, and layer thickness can be estimated within 6÷8 μm. Incorporation of HA maintained this layered morphology (Figure 4C,D), while metal-substituted HA composites (ZnHA and MnHA) demonstrated distinct structural modifications (Figure 4F,I). Although the polymer wall thickness remained unchanged, the macrostructure transitioned to a cellular configuration with ZnHA/MnHA particles localized within cellular compartments. On the other hand, MnHA composites exhibited smaller cell dimensions (pore size = 112 ± 18 μm) and more rounded pore geometries compared to ZnHA counterparts (pore size = 148 ± 23 μm). Indirect porosity assessment was carried out by analyzing the SEM micrographs “Figure 4A,C,F,I,” which are representative of A/P, A/P-HA, A/P-MnHA, and A/P-ZnHA compounds, respectively, by the ImageJ software (ImageJ, ImageJ.net). Semiquantitative comparison between dark (inner pores) and bright (pores contours) areas allowed for the estimation of porosity, expressed as a percentage rate of exposed surfaces: A/P, 56.3%; A/P-HA, 38%; A/P-MnHA, 48.7%; A/P-ZnHA, 50.8%. Although the provided indirect results are roughly estimated, it remains clear that the inclusion of HA (doped or undoped) within the A/P matrix determined a decrease in general porosity. Nevertheless, the presence of Zn and Mn dopants within their respective formulations ensures a higher degree of porosity with respect to the undoped A/P-HA composite.

These findings demonstrate that ZnHA and MnHA incorporation significantly influences scaffold morphology, providing a viable approach for tailoring structural parameters that govern biological performance. The metal-dependent morphological variations suggest potential for targeted modulation of material properties to meet specific clinical requirements.

The observed pore architecture is particularly significant for wound healing applications, as the interconnected cellular structure facilitates (i) efficient exudate absorption, (ii) enhanced blood interaction, and (iii) optimal oxygen delivery to regenerating tissues. Additionally, the high specific surface area of the porous network contributes to rapid hemostatic activity [42,43].

Finally, EDS analysis confirmed homogeneous calcium distribution across all the sponges (Figure 4E,H,K).

**Figure 5:**
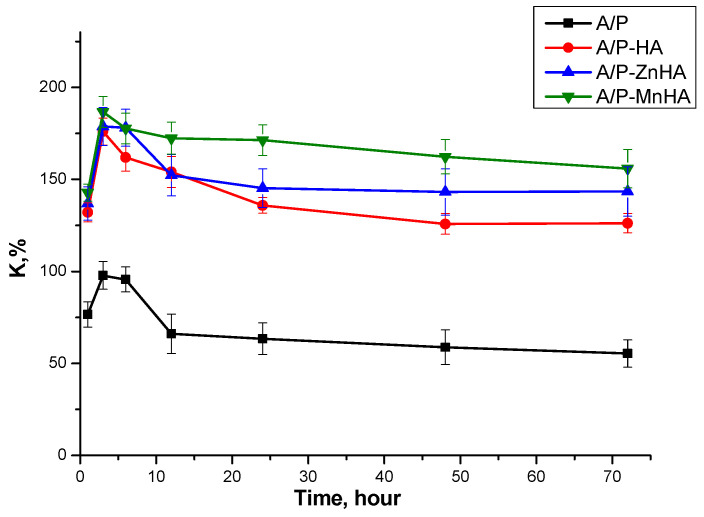
Swelling curves of A/P and A/P-MeHA in physiological solution. The concentration of Me-HA is 0.5%.

Swelling behavior of the sponges was evaluated by measuring mass changes upon immersion in physiological solution (0.9% NaCl). As shown in Figure 5, the pure A/P sponge exhibited the lowest swelling degree. Incorporation of either HA or metal-substituted HA (Me-HA), even at a low concentration (0.5 wt.%), significantly enhanced the swelling capacity. All samples demonstrated rapid swelling during the initial 3 h of immersion, followed by plateau behavior. This kinetic profile suggests dissolution of uncross-linked polysaccharide chains from the sponge matrix into the surrounding medium during the early stages of hydration.

As visible in Figure 5, no significant differences in swelling capacity were observed between HA, ZnHA, and MnHA composites. This indicates that the specific type of calcium phosphate (pristine or metal-substituted) does not substantially influence the swelling behavior of the mineral–polymer sponges. Moreover, although variations in pore size and porosity influence other material properties, they do not appear to affect water absorption characteristics.

These findings suggest that the swelling behavior is primarily governed by the polymeric matrix composition rather than the inorganic phase characteristics or structural parameters of the pore network.

**Figure 6:**
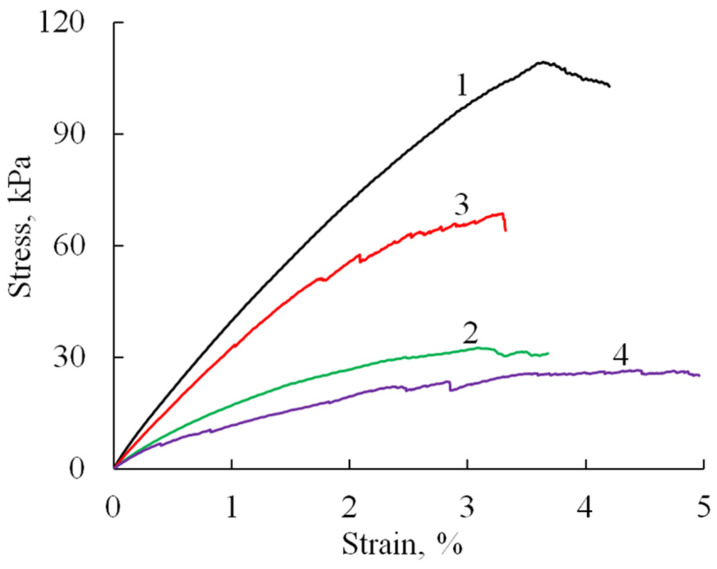
Stress–strain curves (tensile test) of A/P-based samples: A/P (1), A/P containing 0.5 wt.% HA (2), A/P containing 0.5 wt.% Mn-HA (3), A/P containing 0.5 wt.% Zn-HA (4).

The mechanical properties of the prepared sponge materials were investigated testing the uniaxial tension and compression. Tensile tests showed (Figure 6) that the obtained materials (A/P without phosphate) have quite good strength characteristics (Table 1), with an elastic modulus of about 5 MPa, tensile elongations of about 4%, and a strength that reaches values up to 100 kPa.

**Figure 7:**
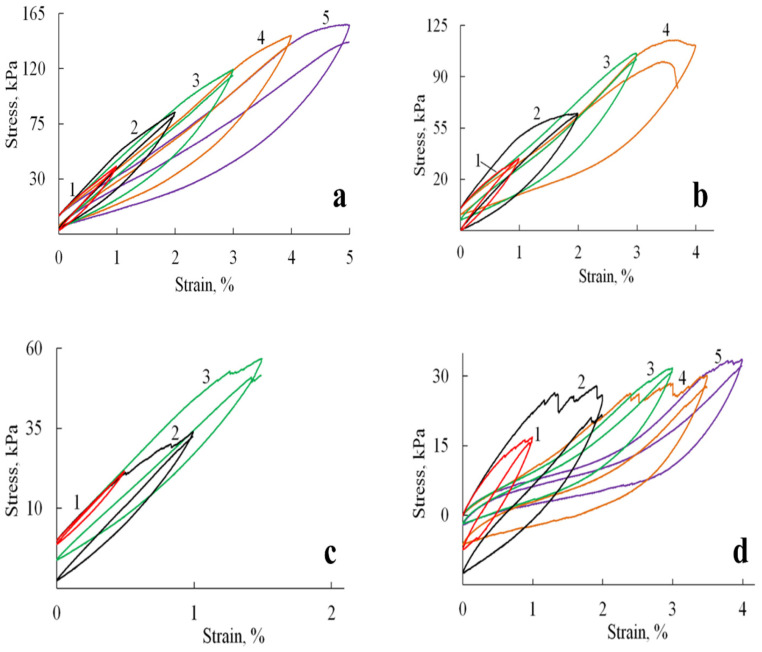
Hysteresis loops formed by stress-strain curves during cyclic loadings for A/P based samples: (**a**) A/P sponge; (**b**) A/P sponge containing 0.5 wt.% HA; (**c**) A/P sponge containing 0.5 wt.% MnHA; (**d**) A/P sponge containing 0.5 wt.% ZnHA.

The introduction of HA and substituted HA led to a decrease in the elastic modulus of the material by about two times and strength by 30–70%, which may be due to changes in the microstructure of the samples (as indicated by SEM data), an increase in porosity and pore size, as well as defects in the formation of sponges in the presence of filler particles. At the same time, it should be noted that the failure of samples containing HA likely occurs through the progressive breakdown of pore walls and the formation of microcracks. This gradual degradation is evidenced by the appearance of stress-relief features on the tensile curves (Figure 7b–d).

Cyclic loading tests revealed distinct mechanical responses between the pure A/P sponge and HA-incorporated composites. As shown in Figure 7a, the A/P sponge exhibited stable mechanical properties characterized by fully reversible deformation up to the point of fracture. The hysteresis loops retained consistent shapes throughout successive deformation cycles, although each subsequent cycle exhibited a slight reduction in stretching stress compared to the initial cycle, an observation consistent with mechanical softening behavior in polymeric materials.

The incorporation of HA significantly modified the mechanical response under cyclic loading conditions (Figure 7b–d). The composite materials displayed evolving hysteresis loop morphologies, with observable discontinuities suggesting progressive microstructural changes. These alterations likely result from the gradual development of microcracks and partial pore wall failure during cycling, which modifies the sponge′s primary structure and consequently its mechanical behavior. Despite these microstructural changes, the composites preserved their macroscopic integrity and maintained reproducible viscoelastic behavior throughout testing.

**Figure 8:**
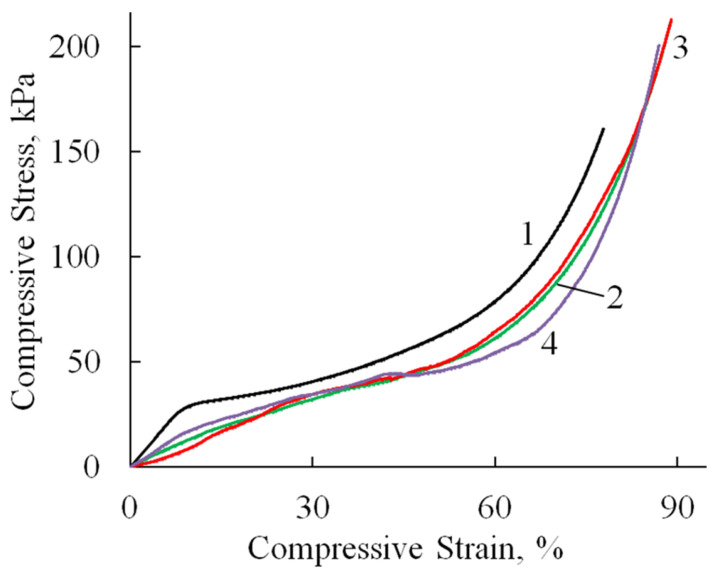
Stress–strain curves (compression test) of A/P (1), A/P containing 0.5 wt.% HA (2), A/P containing 0.5 wt.% MnHA (3), and A/P containing 0.5 wt.% ZnHA (4).

Compression tests revealed consistent deformation patterns across all specimens (Figure 8, Table 2). An initial linear elastic region extended to 10–36% strain, followed by a plateau phase with minimal stress increase corresponding to pore wall bending. At 60–70% strain, the materials entered a densification phase characterized by structural collapse and rapid stress elevation due to pore wall failure and compaction. All samples withstood deformations approaching 100% strain without complete failure. It is worth noting that the compressive modulus values were approximately an order of magnitude lower than the corresponding tensile moduli (Table 2), reflecting the inherent mechanical anisotropy of these porous structures. This mechanical profile indicates that while HA incorporation influences cyclic stability, the composites retain sufficient structural integrity for applications requiring compressibility and energy absorption.

**Figure 9:**
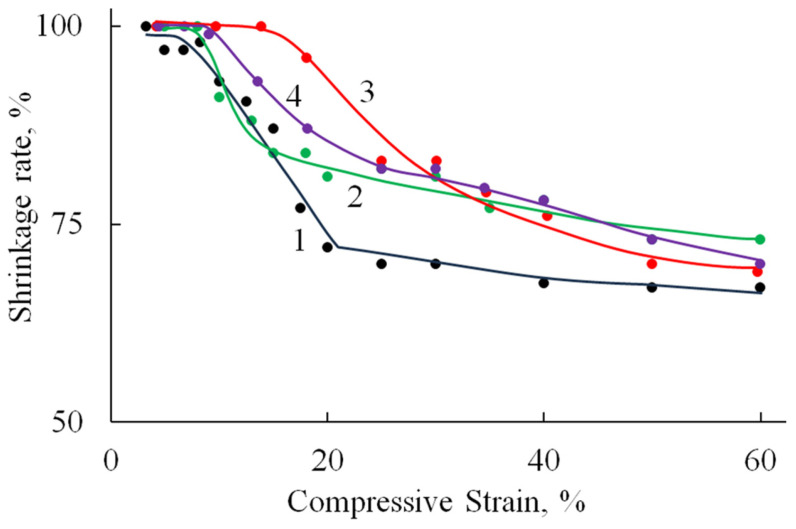
The dependence of the fraction of reversible deformation on the compression strain (under cyclic compressive loading) of A/P (1), A/P containing 0.5 wt.% HA (2), A/P containing 0.5 wt.% MnHA (3), and A/P containing 0.5 wt.% ZnHA (4).

Figure 9 shows the relationship between the reversible strain fraction and the compression ratio, as determined under cyclic compression–recovery conditions.

Deformation is fully reversible up to a compression level of 9–15%, corresponding to the elastic limit region (Table 2). Beyond this range, the proportion of reversible deformation rapidly decreases to approximately 70%, a value that remains stable up to 60% compression. At higher compression levels, compaction of the porous structure begins, involving significant structural reorganization and the breakdown of pore walls.

The incorporation of HA, both in its undoped form and doped with Zn ions, into the polymer matrix leads to a reduction in sample deformation under identical loading conditions. A less pronounced effect is observed with the addition of MnHA, where the recorded strain values are comparable to those of the A/P sample.

**Figure 10:**
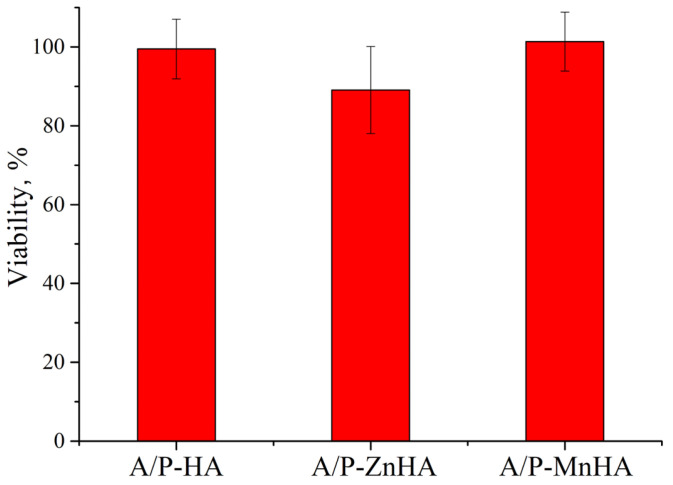
MTT viability assay of NCTC L929 cells in incubation with sample extracts for 24 h. Viability is reported as % ± SD.

The metabolic activity of NCTC L929 cells in the presence of A/P with HA, A/P with ZnHA, and A/P with MnHA extracts was investigated using the MTT assay. As reported in Figure 10, the presence of A/P-ZnHA sponge extracts induces a decrease in cell viability when compared to the control.

The observed cellular responses are coherent with previous findings reporting cytotoxic effects in zinc ion-cross-linked alginate films [44].

**Figure 11:**
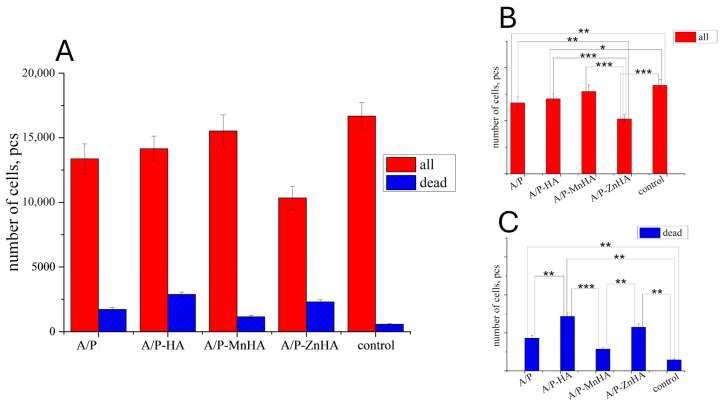
Number of DPSC cells ±SD (total—red bars; dead—blue bars) in presence of the developed materials after 24 h of cultivation (**A**). The p values (one-way ANOVA test) between groups are reported for both the total number of cells (**B**) and the number of dead cells (**C**): *, *p* < 0.05; **, *p* < 0.01; ***, *p* < 0.001.

However, the results obtained on the DPSC viability test revealed only a marginal decrease in metabolic activity when cultured with the developed materials (Figure 11). This behavior can be attributed to the diffusion limitations within the three-dimensional culture system. Alginate–pectin sponges incorporating MnHA exhibited the most favorable cytocompatibility profile, showing minimal deviation from control conditions, fostering a correlation with their optimized pore architecture. Among the investigated samples, only MnHA showed no significant difference from the control in the total number of adhering cells (*p* > 0.05, Mann–Whitney test). However, all tested samples exhibited a significant increase in the number of nonviable cells compared to the control.

**Figure 12:**
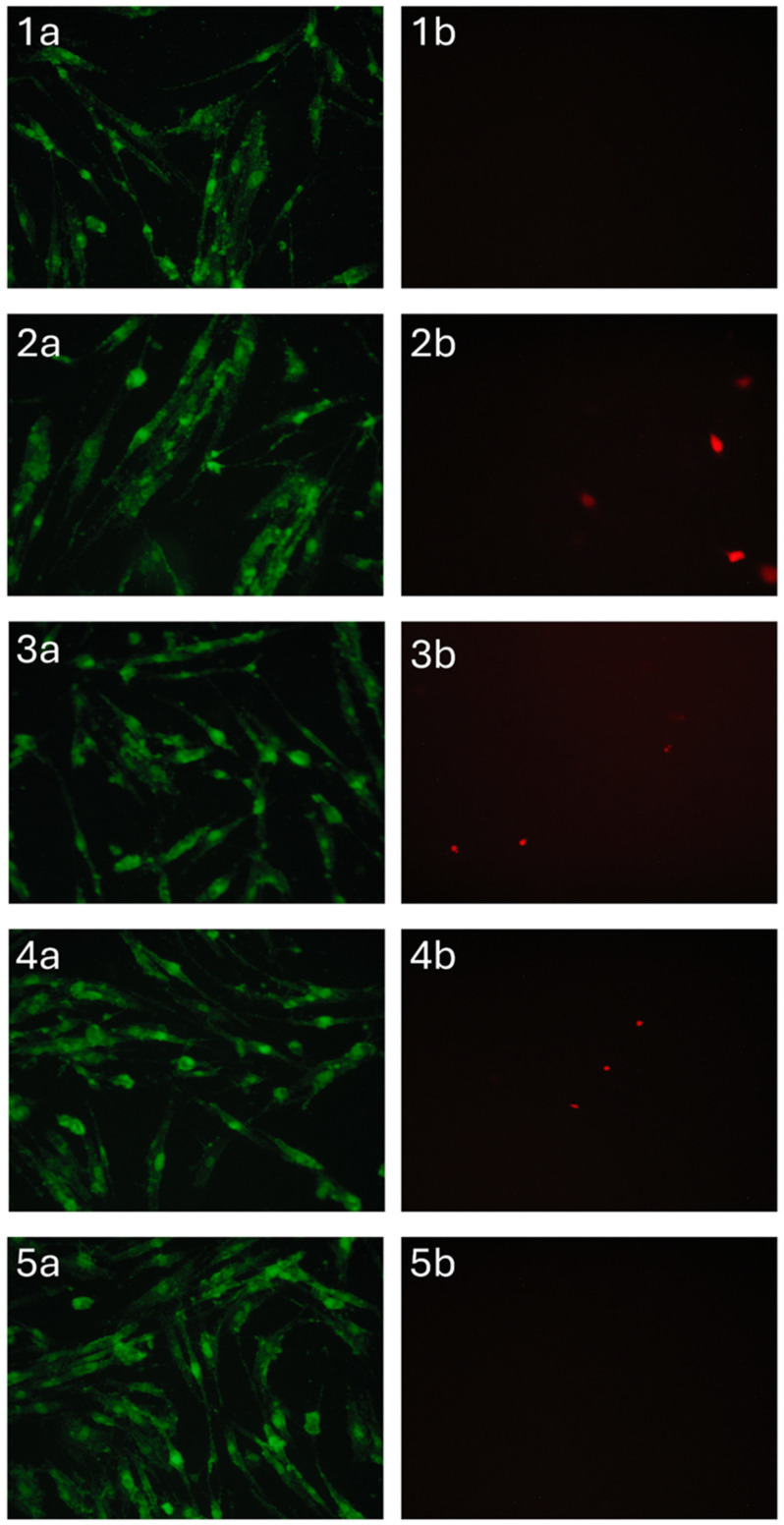
DPSC cells cultured for 24 h in the presence of A/P (1), A/P-HA (2), A/P-MnHA (3), A/P-ZnHA (4), control (5). SYTO 9 (a) and PI (b) staining.

Materials containing zinc-substituted HA demonstrated a modest suppressive effect on cell viability, potentially mediated through medium acidification resulting from material hydrolysis. Importantly, morphological analysis confirmed the maintenance of normal DPSC morphology across all test groups (Figure 12). The limited presence of propidium iodide-stained nuclei further substantiates the absence of significant cytotoxic effects, suggesting overall biocompatibility of the developed sponge materials. These findings indicate that while compositional variations influence cellular responses, none of the tested formulations exhibited detrimental effects sufficient to compromise their potential biomedical applications.

The antibacterial activity of the composite sponges against the Gram-positive bacteria strain *B. atrophaeus B-723 (1)* and a rhizosphere Gram-negative bacteria strain *P. protegens P4 (2)* was investigated in vitro.

**Figure 13:**
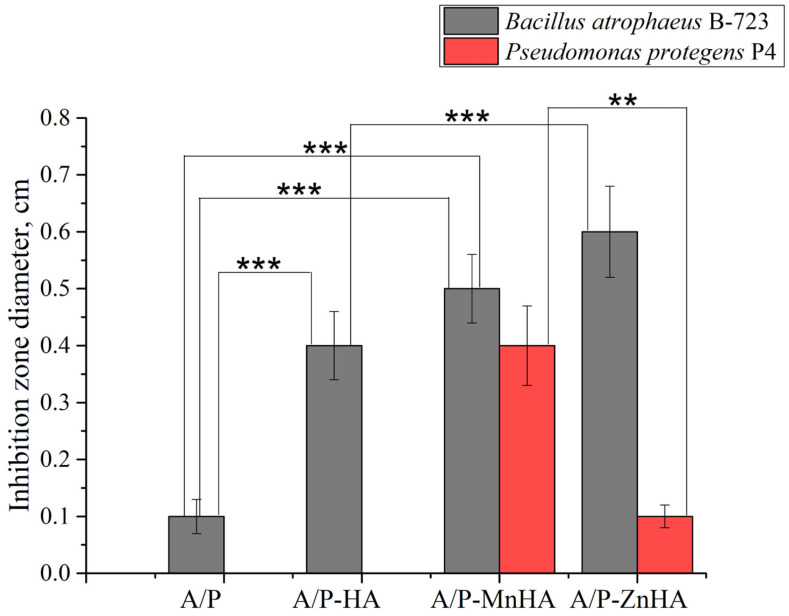
Diameter ±SD of the inhibition zone of A/P sponges and composite porous materials based on A/P matrix with HA, MnHA and ZnHA. Inhibition zones are reported for *B. atrophaeus B-723* strain (grey bars) and *P. protegens P4* strain (red bars). Statistical significance between groups (one-way ANOVA test) is reported: **, *p* < 0.01; ***, *p* < 0.001.

As shown in Figure 13, the composite sponges containing MnHA and ZnHA exhibited pronounced antibacterial activity against the Gram-positive strain *B. atrophaeus* B-723. The MnHA-containing sponge also displayed a clear and measurable inhibition zone against the Gram-negative bacterium *P. protegens P4*, whereas the ZnHA composite showed only a minimal effect. The control sponge containing unsubstituted HA exhibited antibacterial activity, with a narrow inhibition zone, against *B. atrophaeus* and no observable effect against *P. protegens*. The differences in inhibition zone diameters highlight the broad-spectrum bactericidal efficacy conferred by Mn^2+^ substitution in HA, and to a lesser extent, by Zn^2+^ substitution. A/P and A/P-HA biocomposites revealed a higher antibacterial activity against the Gram-positive bacteria compared to Gram-negative strain.

## 4. Conclusions

In the present work, three-dimensional porous composites were successfully synthesized through the integration of plant-derived polysaccharides (sodium alginate and pectin) with Zn- or Mn-substituted HAs via lyophilization and calcium salt cross-linking. Structural characterization by XRD and FTIR confirmed the single-phase apatite structure of ZnHA and MnHA, with crystallite sizes below 1 µm. Mechanical testing revealed that the pristine alginate–pectin sponges exhibited an elastic modulus of 4.7 MPa and tensile strength of 0.10 MPa, while HA incorporation reduced these properties by 30–70%, attributed to increased porosity (pore sizes: 112 ± 18 µm for MnHA vs. 148 ± 23 µm for ZnHA) and microstructural defects.

SEM analysis demonstrated that ZnHA and MnHA incorporation transformed the layered A/P matrix (layer thickness < 100 µm) into a tridimensional architecture, containing mineral particles. Swelling studies in physiological solution revealed a 2.3–2.8-fold increase in water uptake for HA-containing composites (125–155%) compared to pure A/P sponges (55%), governed primarily by the polymeric matrix rather than inorganic phase composition.

The biological tests confirmed the cytocompatibility of the developed sponges, with A/P-MnHA composites showing minimal impact on the DPSC viability, whereas A/P-ZnHA induced moderate suppression, likely due to hydrolysis-mediated acidification.

The antibacterial assays demonstrated that A/P-MnHA composites exhibited significant inhibition zones against both Gram-positive *B. atrophaeus* and Gram-negative *P. protegens*, while A/P-ZnHA showed pronounced efficacy specifically only against the *B. atrophaeus* strain. These bactericidal effects are attributed to Mn^2+^/Zn^2+^ substitution in HA.

The porous microstructure, enhanced swelling kinetics, and antibacterial effectiveness constitute optimal preliminary characteristics for the use of the developed sponges as resorbable matrices for biomedical applications.

While the presented results offer valuable insights into the effects of a composite composition on microstructure, mechanical properties, swelling behavior, biocompatibility, and antibacterial activity, the broader applicability of these findings may be limited by the relatively small number of biological trials conducted. Future studies will focus on investigating the interactions of porous, bioresorbable A/P-based mineral–polymer composites with various mammalian cell types both in vitro and in vivo, along with an expanded range of microbiological assays.

## Figures and Tables

**Table 1:** Mechanical characteristics of the A/P sponge samples determined by tensile test.

Sample	Modulus of Elasticity, MPa	Strength, MPa	Breaking Elongation, %
A/P	4.7 ± 0.5	0.10 ± 0.02	3.5 ± 1.0
A/P + 0.5% HA	2.5 ± 0.2	0.07 ± 0.01	5.5 ± 1.5
A/P + 0.5% MnHA	4.2 ± 0.4	0.06 ± 0.01	2.5 ± 1.0
A/P + 0.5% ZnHA	2.5 ± 0.3	0.03 ± 0.01	4.5 ± 1.5

**Table 2:** Mechanical characteristics of the A/P sponge samples determined by uniaxial compression.

Sample	Region of Elastic Behavior		Limit		Sealing	
	Modulus, MPa	Elastic Limit Def., %	Def., %	Stress, kPa	Def., %	Stress, kPa
A/P	0.34 ± 0.04	8.5 ± 0.5	9 ± 2	28.2 ± 0.3	60 ± 4	79.1 ± 0.8
A/P + 0.5% HA	0.16 ± 0.02	8.5 ± 0.5	36 ± 3	37.3 ± 0.4	70 ± 5	87.9 ± 0.9
A/P + 0.5% MnHA	0.17 ± 0.02	15 ± 1.5	25 ± 2	32.1 ± 0.3	70 ± 5	97.8 ± 1.0
A/P + 0.5% ZnHA	0.19 ± 0.03	10 ± 1.0	13 ± 2	20.7 ± 0.2	73 ± 6	86.6 ± 0.9

## Data Availability

The experimental data on the results presented in this manuscript are available upon formal request to the corresponding author.
